# Treatment of Osteoarthritis of the Knee with a Combination of Autologous Conditioned Serum and Physiotherapy: A Two-Year Observational Study

**DOI:** 10.1371/journal.pone.0145551

**Published:** 2015-12-28

**Authors:** Jaime Baselga García-Escudero, Pedro Miguel Hernández Trillos

**Affiliations:** Hospital Ruber International, Madrid, Spain; University of Texas Health Science Center at Houston, UNITED STATES

## Abstract

**Background:**

Autologous conditioned serum (ACS) is an autologous blood product that has shown efficacy against knee osteoarthritis (OA) in randomized controlled trials. However, there are few reports of its effectiveness in everyday practice. Here, we report clinical efficacy results from a two-year prospective observational study of patients with highly symptomatic knee OA who received ACS in conjunction with physiotherapy.

**Methods:**

118 patients with unilateral knee OA (Kellgren-Lawrence grades I–IV), who were candidates for surgery but instead chose conservative treatment, were treated with a combination of four intra-articular injections of ACS (2 mL each) once weekly over four weeks and subsequent physiotherapy applied 4 weeks after ACS injection. Main endpoints of the study were pain (Numeric Rating Scale [NRS]) assessed at 0, 3, 6, 12 and 24 months, and Western Ontario and McMaster Universities Osteoarthritis Index (WOMAC) global score, assessed at 0 and 24 months. The effect size (Cohen’s d) was calculated for pain and WOMAC outcomes, with effect sizes >0.8 considered large.

**Results:**

By 3 months, there were significant improvements in pain (NRS) from baseline (-63.0%, p<0.001), which were maintained over 24 months. Mean WOMAC global score was reduced at 24 months compared to baseline (-56.9%, p<0.001), as were WOMAC subscores of pain (-86.0%, p<0.001) and function (-51.3%, p<0.001). Effect sizes for pain (>5) and WOMAC improvement (8.0–13.6) were very large. Only one patient received total knee joint replacement during the study. Clinical improvement did not correlate with gender, age, Kellgren-Lawrence grade, or body mass index.

**Conclusions:**

Treatment with ACS and physiotherapy produced a rapid decline in pain, which was sustained for the entire two years of the study. This was accompanied by a large improvement in WOMAC scores at two years. These results confirm that ACS combined with physiotherapy is an effective treatment for OA of the knee.

## Introduction

Osteoarthritis (OA) is a common, incurable and difficult to treat disease. Joint surgery is an option in progressed cases [1]; however, patients often request less invasive alternatives. As the disease affects a limited number of joints and is not known to have important systemic sequelae, there is much interest in intra-articular therapy, where the drug is injected directly into the affected joint [2]. Hyaluronic acid (HA), corticosteroids and platelet-rich plasma are all used in this context, but none of these therapies produce dramatic, lasting improvement [1, 3].

Autologous conditioned serum (ACS) is an alternative intra-articular therapy for the treatment of OA. ACS is derived from autologous blood by incubating venous blood for six hours in a specially designed syringe containing glass beads. Exposure of blood to the beads induces peripheral blood leukocytes to produce increased amounts of anti-inflammatory cytokines such as IL-1Ra [4]. The post-incubation serum is recovered and delivered to affected joints, usually in a series of 3–6 intra-articular injections [4]. There have been two randomized, controlled trials (RCTs) of ACS in patients with OA of the knee and in both studies patients reported improvements in the clinical signs and symptoms of OA [5, 6].

There have, however, been few reports of the experience of physicians using ACS in orthopedic practice outside the rigorous confines of an RCT. Here, we present results from an independent, investigator-initiated, prospective, observational study of ACS treatment in combination with physiotherapy in knee OA outpatients. As these patients had highly symptomatic disease, they were candidates for surgical intervention, but instead elected to undergo conservative treatment. The objective of this study was to evaluate the clinical symptoms in these patients following two years of ACS treatment combined with subsequent physiotherapy compared to baseline.

## Methods

### Patients

This two-year, single-site, prospective observational study was conducted from 2009 to 2011 and included patients ≥18 years of age with OA Kellgren-Lawrence grade I–IV and painful OA of the knee (numeric rating scale [NRS] ≥6). X-ray based grading for knee OA was performed by two independent observers according to the Kellgren-Lawrence criteria. Patients were only included in the study if they preferred conservative treatment to possible surgery and agreed to a follow-up period of 2 years. Exclusion criteria included pregnancy, neurological disorders, joint infection, spondyloarthropathies, gout, hyperlipidemia, rheumatoid arthritis, sarcoidosis or pathologies of the lower limb which would interfere with the evaluation of knee OA. Patients were also excluded if they had received intra-articular injection(s) or Symptomatic Slow-Acting Drugs in OsteoArthritis (SYSADOA) in the past two months, oral corticoid treatment in the last month, or oral NSAID treatment or topical corticoid treatment in the past week. All patients agreed to anonymous data analysis and publication by signing an informed consent form before treatment. This study was approved by the Ethics Committee of Clinical Investigation, Hospital Ruber Internacional, Madrid, Spain, and was conducted in conformance with this decision.

### Treatments

#### ACS preparation and application

For ACS therapy, 4 x 10 mL of blood was collected from each patient using four EOT^®^II syringes (Orthogen Lab Services GmbH, Düsseldorf, Germany) and incubated at 37°C for 6 hours. Subsequently, the blood was centrifuged at 3000 g for 10 minutes. The supernatant conditioned serum was collected, filtered through a 0.22 μM syringe tip filter (Millex GP, Merck Millipore, Tullagreen, Carrigtwohill, Cork, Ireland) and either injected intra-articularly or frozen at -20°C until clinical use. Patients received four intra-articular injections of 2 mL ACS once weekly over four weeks.

#### Physiotherapy

Patients received a uniform rehabilitation program starting four weeks after the last injection of ACS which consisted of three 50 minute rehabilitation sessions per week for 10 weeks, 30 sessions in all. Physiotherapy consisted of a combination of physiotherapy and transcutaneous electric nerve stimulation (TENS). Rehabilitation included kinesiotherapy, muscular strengthening, joint range of motion exercises and TENS.

### Outcome Measurements

Clinical outcome data were collected during the 24 month observation period. Clinical examinations and pain (numeric rating scale [NRS]) were recorded at 0, 3, 6, 12 and 24 months, with “0” indicating no pain and “10” indicating worst pain. The Western Ontario and McMaster Universities Osteoarthritis Index (WOMAC) score was recorded at 0 and 24 months. WOMAC is a patient-administered, quality-of-life instrument validated for the assessment of patients with OA [7]. The WOMAC questionnaire consists of 24 questions; Q1–Q5 relate to pain (WOMAC pain), Q6–Q7 relate to stiffness (WOMAC stiffness) and Q8–Q24 relate to function (WOMAC function). Each question was graded on a 0–4 Likert scale with “0” indicating no symptoms, and “4” indicating worst symptoms. WOMAC global score was calculated as the sum of the three subscores, which ranges from 0–96.

### Statistical Analysis

Data were tabulated using MS Excel, which was also used to produce basic statistics and graphs. T-tests (2-sided) and effect sizes were computed using programs written in Visual Basic for Applications by S. Cleveland (University of Düsseldorf). A p-value <0.05 was considered significant. The effect size (Cohen’s d) is a dimensionless measure of change due to treatment and is independent of sample size. It is computed as the difference between groups and pre- and post-records, divided by the combined standard deviation of these data sets. Effect sizes >0.8 were considered large.

## Results

### Patient Characteristics

In total, 118 routine outpatients (75 female, 43 male) were enrolled in the study. The mean age at baseline was 59.1 (range 34–81); full baseline demographics are given in Table 1. These patients were highly symptomatic, with an average pain (NRS) score of 8.1 and high scores for WOMAC global, WOMAC pain and WOMAC function (Table 1). The Kellgren-Lawrence score averaged 2.8 and ranged from a score of 1 (minimal joint space narrowing) to 4 (bone on bone). The majority of patients had grade 3 OA (80 patients, 68%). Due to the highly symptomatic nature of their OA, patients entered the clinic expecting to receive surgery.

**Table 1:** Baseline demographics and characteristics.

Baseline characteristics	ACS/PT patients (n = 118)	
	Mean^a^	Range
Age (years)	59.1	34–81
Gender (female/male)	75/43	N/A
BMI	29.6	22–41
Affected knee (left/right)	25/93	N/A
OA grade	2.8	1–4
NRS (0–10)	8.1	6–10
WOMAC global (0–96)	81.6	75–90
WOMAC pain (0–20)	17.9	15–20
WOMAC stiffness (0–8)	3.4	1–8
WOMAC function (0–68)	60.4	51–67

^a^Mean given for all baseline characteristics except for gender and affected knee, where n numbers are presented. ACS: Autologous conditioned serum; BMI: Body Mass Index; OA: Osteoarthritis; PT: Physiotherapy; NRS: Numeric Rating Scale; WOMAC: Western Ontario and McMaster Universities Osteoarthritis Index.

### Efficacy

**Fig 1:**
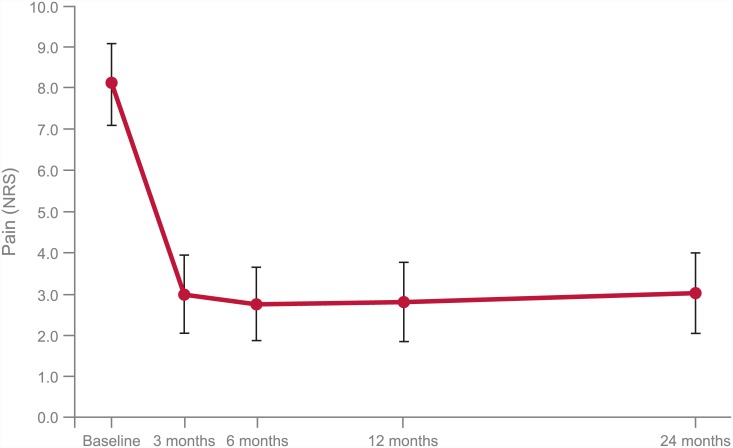
Pain (NRS) scores for baseline, 3, 6, 12 and 24 months after treatment with ACS and physiotherapy. Error bars denote standard deviation. ACS: Autologous conditioned serum; NRS: Numeric Rating Scale.

**Fig 2:**
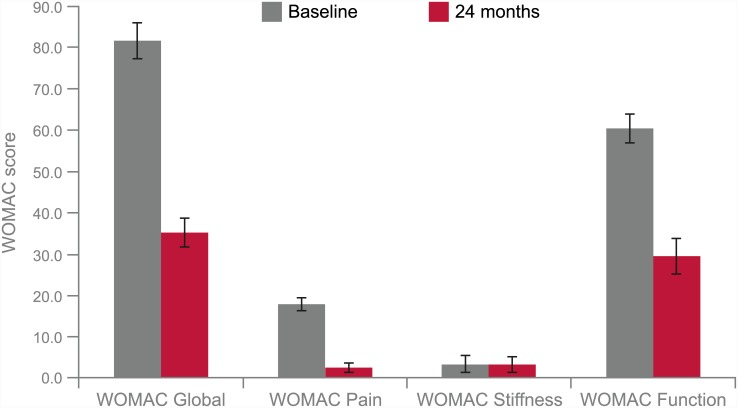
WOMAC global and WOMAC subscores pain, stiffness and function at baseline and 24 months after treatment with ACS and physiotherapy. Error bars denote standard deviation. ACS: Autologous conditioned serum; WOMAC: Western Ontario and McMaster Universities Osteoarthritis Index.

Mean pain (NRS) improvements after 3, 6, 12 and 24 months were statistically significant with decreases of -63%, -66%, -65% and -63%, respectively, compared to baseline (p<0.001). Improvements in pain scores observed at 3 months were maintained in the follow-ups up to 24 months (Table 2, Fig 1). The effect size for pain improvement was greater than 5 (Table 2). By the end of the study, there were significant improvements in WOMAC global, pain and function scores, with decreases of -56.9%, -86.0% and -51.3% at 24 months compared to baseline, respectively (p<0.001, Fig 2 and Table 3). The effect sizes ranged from 8.0–13.6 (Table 3). However, there was no significant improvement in the WOMAC subscore stiffness (Table 3).

**Table 2:** Mean pain (NRS) scores at baseline, 3, 6, 12 and 24 months.

	ACS/PT patients (n = 118)		
Timepoint	Mean pain (NRS) score (range)	p-value compared to baseline	Effect size d compared to baseline
Baseline	8.10 (6–10)	-	-
3 months	3.00 (1–5)	p<0.001	d = 5.3
6 months	2.76 (0–4)	p<0.001	d = 5.6
12 months	2.81 (0–5)	p<0.001	d = 5.4
24 months	3.03 (0–4)	p<0.001	d = 5.1

P-values and effect sizes d are given in comparison to baseline.ACS: Autologous conditioned serum; PT: Physiotherapy; NRS: Numeric Rating Scale.

**Table 3:** WOMAC global and WOMAC subscores at baseline and 24 months.

**WOMAC global: Stratification by Kellgren-Lawrence OA grade**						
**OA Grade**	**n**	**Baseline Mean (range)**	**24 months Mean (range)**	**% change**	**p-value**	**Effect size d**
OA grade I	13	81.2 (78–84)	34.9 (30–40)	-57.0	p<0.001	11.3
OA grade II	13	83.3 (75–90)	34.7 (30–42)	-58.3	p<0.001	10.8
OA grade III	80	81.5 (76–90)	35.3 (30–42)	-56.7	p<0.001	11.9
OA grade IV	12*	81.3 (76–90)	35.5 (30–42)	-56.3	p<0.001	13.6
**WOMAC global and WOMAC subscores**						
**Score**	**n**	**Baseline Mean (range)**	**24 months Mean (range)**	**% change**	**p-value**	**Effect size d**
WOMAC global	118	81.6 (75–90)	35.2 (30–42)	-56.9	p<0.001	11.8
WOMAC pain	118	17.9 (15–20)	2.5 (0–5)	-86.0	p<0.001	11.5
WOMAC stiffness	118	3.36 (1–8)	3.3 (1–8)	-3.6	p>0.6	0.006
WOMAC function	118	60.4 (51–67)	29.4 (21–39)	-51.3	p<0.001	8.0

*The values of the patient with OA grade IV who elected for total knee endoprosthesis after 12 months were included at 24 months using LOCF imputation. ACS: Autologous Conditioned Serum; PT: Physiotherapy; WOMAC: Western Ontario and McMaster Universities Osteoarthritis Index.

Clinical effect as measured by pain outcome did not appear to correlate with gender, age, Kellgren-Lawrence OA grade, or BMI (S1 Fig). A trend towards more pronounced improvement was noted in patients 30–39 years old (n = 6) with less pronounced improvement observed for patients 80–89 years old (n = 3). However, the small number of cases in these two groups did not allow for reliable statistical analysis (Table 4).

**Table 4:** Stratification of clinical outcome by patient age range.

			Pain (NRS) score		WOMAC score	
Age range	n	Mean OA grade	Baseline	24 months	Baseline	24 months
30–39	6	2.50 (1–4)	8.50 (6–10)	2.83 (0–4)	81.17 (76–90)	34.00 (30–39)
40–49	12	3.08 (3–4)	7.67 (7–10)	3.33 (2–5)	82.00 (77–90)	35.08 (30–42)
50–59	44	2.73 (1–4)	8.16 (7–10)	2.95 (1–4)	80.86 (76–90)	35.32 (30–42)
60–69	37	2.76 (1–4)	8.00 (6–10)	3.00 (2–4)	82.78 (75–90)	34.78 (30–42)
70–79	16	2.69 (1–4)	8.38 (7–10)	3.00 (1–4)	80.94 (75–90)	35.38 (32–42)
80–89	3	3.33 (3–4)	8.00 (-)	3.67 (2–5)	80.67 (78–85)	40.00 (39–42)

Data are mean (range) unless otherwise stated. OA: Osteoarthritis; NRS: Numeric Rating Scale; WOMAC: Western Ontario and McMaster Universities Osteoarthritis Index

### Surgical Interventions

Only one patient was lost to follow-up. This individual entered with a Kellgren-Lawrence score of IV and a pain (NRS) score of 8 which improved to 4 at 6 months and was recorded as 5 at 12 months. Despite reporting improvements in pain, this patient elected to receive a total knee endoprosthesis after 1 year of treatment. Data for this patient at 12 months were applied to the 24 month time-point using last observation carried forward (LOCF) imputation. All other patients completed the 24-month study without requiring surgery. ACS was well tolerated, with no new safety signals detected in this study.

## Discussion

ACS has shown promise in RCTs as a local therapy for OA of the knee [5, 6], but there are few reports of efficacy in everyday practice. In this observational study of ACS combined with physiotherapy, pain scores declined by over 60% during the first 3 months after treatment and remained low for the remainder of the study. WOMAC scores also improved dramatically, and after 24 months showed reductions of nearly 57% for global, 51% for function and a striking reduction of 86% for pain. For all of these outcomes, effect sizes were very high. Only the WOMAC stiffness component, which was modest at the outset of the study, remained unchanged. This study confirms a significant and sustained response to ACS in a clinical setting when combined with physiotherapy.

It is interesting to speculate that the large degree of improvement seen here reflects the highly symptomatic nature of the patients’ OA when entering the study. It is also noteworthy that strong symptomatic improvement extended across all grades of OA, including those with the most severe disease. This observation agrees with findings by Baltzer *et al*. who reported excellent improvement in pain in OA of the hip with little correlation to radiologic staging of the disease [8]. Age, gender and BMI also did not correlate with outcome, suggesting ACS treatment is effective in patients independent of these factors and may thus be used in patients for whom certain drugs might be contraindicated. Controlled studies focusing on advanced-stage OA with strong symptoms are recommended to confirm these findings.

This study did not address whether the combination of ACS and physiotherapy influenced joint degeneration, and therefore might be disease-modifying. It also made no attempt to determine the precise mode of action of ACS, which is still under investigation. In joints affected by OA, chondrocytes and synovial cells produce increased levels of pro-inflammatory cytokines such as IL-1, which contribute to the destruction of articular cartilage [9]. The balance of catabolic and anabolic cytokines in OA is therefore altered, disrupting normal joint homeostasis [10]. ACS contains enhanced concentrations of both anti-inflammatory cytokines and growth factors such as IL-1Ra, IL-10, TGFß, PDGF, HGF and IGF [4], which are known to be involved in natural wound-healing processes. Previous studies have also found evidence for a regenerative action of ACS therapy across various indications, both in animals and humans [11–16]. The high concentration of anti-inflammatory and growth-factor molecules found in ACS likely explains the striking clinical effect observed in this study. Furthermore, the combination of synergistic molecules in ACS may help re-establish a healthy joint homeostasis, contributing to the sustained effect observed two years following ACS treatment.

The improvements in pain and WOMAC score reported in this study are supported by data from RCTs of ACS in knee OA [5, 6]. In the first RCT conducted by Baltzer *et al*., patients with knee OA were randomised to ACS, HA, or intra-articular placebo. In this study, ACS was shown to be significantly superior compared to HA and placebo for all efficacy outcome measures and time points [5]. Following ACS treatment, significant reductions in pain (VAS) and WOMAC score were observed, which were of a similar magnitude to results reported here: at Week 26, the paper reports a nearly 60% reduction in VAS score compared to baseline and over 50% reduction in WOMAC Global score. Improvements in pain and WOMAC score were sustained over two years [5], in line with the long-term effect of ACS observed in the present study. In the second RCT by Yang *et al*., improvements in WOMAC and VAS scores were also observed with ACS treatment, but these were smaller (approximately 20% for pain and 19% for WOMAC at 12 months compared to baseline) and were not significantly different to placebo [6]. There were some methodological limitations to the study by Yang *et al*., however, which limit the conclusions that can be drawn from these results. These include the unknown analgesic dose use during the study and low disease severity at enrolment.

Direct comparison of ACS with other treatments for OA is challenging due to differences in study design, conflicting clinical trial results and the lack of head-to-head studies. Although total knee arthroscopy is often considered the final treatment option for patients with knee OA, a number of studies have suggested that arthroscopic surgery does not provide any additional benefit for patients with OA [17–19]. The evidence base for some intra-articular therapies is similarly unclear. Intra-articular corticosteroids have been shown to provide short-term pain relief in OA [20], though concerns regarding cartilage damage with longer treatment durations limit their usefulness [21]. HA intra-articular injection has been widely used for knee OA, yet its efficacy is debated, with meta-analyses of clinical trials reaching different conclusions [22, 23]. Efficacy outcomes for the autologous blood product platelet-rich plasma are also uncertain [24], which may be partly due to variations in preparation methods [25]. Although few RCTs have been conducted to date, the available evidence suggests that ACS may be a promising conservative treatment option for knee OA, with the advantage of a standardized preparation method.

A major limitation of this study is the lack of control group, as this study was planned and performed as a prospective observational study. This makes it difficult to assess whether these patients’ symptoms improved due to the addition of ACS to physiotherapy, or whether their symptoms improved due to physiotherapy alone. However, in our experience, physiotherapy alone produces a pain improvement of about 25% (data not shown). In addition, up to 35% primary improvement has been described for intra-articular placebo injections in trials of OA [26]; placebo effects for intra-articular treatment are usually higher than for oral placebo therapy. An additional limitation was that treatments in this study were administered under routine conditions with no blinding; therefore the results may be affected by doctor and patient bias and also influenced by patient self-selection effects. Despite these limitations, the data presented here support previously reported results from studies of ACS in knee OA and provide important information on the efficacy of ACS combined with physiotherapy in a real-world setting.

## Conclusions

Combination therapy of ACS followed by physiotherapy significantly reduced OA symptoms compared to baseline in a real life outpatient cohort with severe osteoarthritic knee pain, independently of disease stage. The authors consider the combination of ACS and physiotherapy as a modality that can improve quality of life in OA patients and may postpone joint surgery.

## Supporting Information

S1 Fig Pain (NRS) stratified by A) Gender B) Age C) OA grade and D) BMI. (PDF)

S1 Table Stratification of WOMAC global by pain (NRS) outcome. (DOCX)
