# Supplementary figures and images for: Treatment of Osteoarthritis of the Knee with a Combination of Autologous Conditioned Serum and Physiotherapy: A Two-Year Observational Study
Source: PLoS One. 2015 Dec 28;10(12):e0145551. doi: 10.1371/journal.pone.0145551 (PMC4692499; doi:10.1371/journal.pone.0145551)

**A**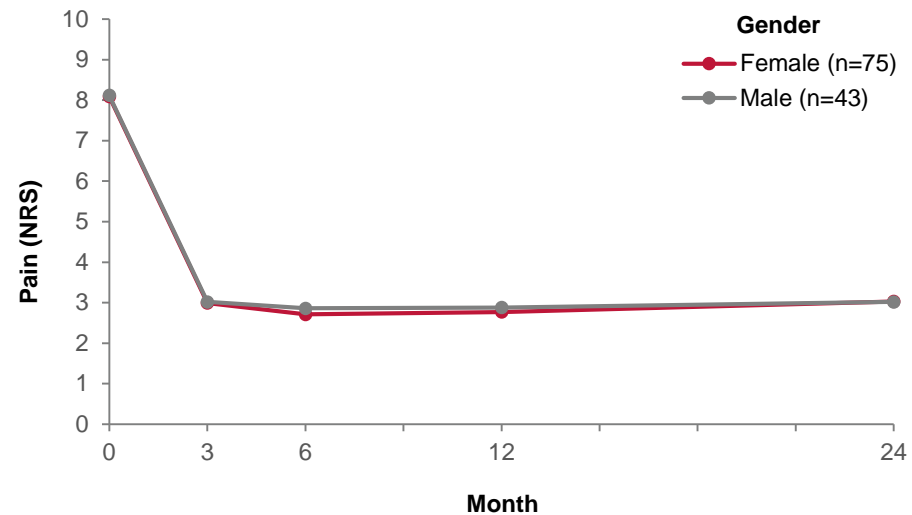**B**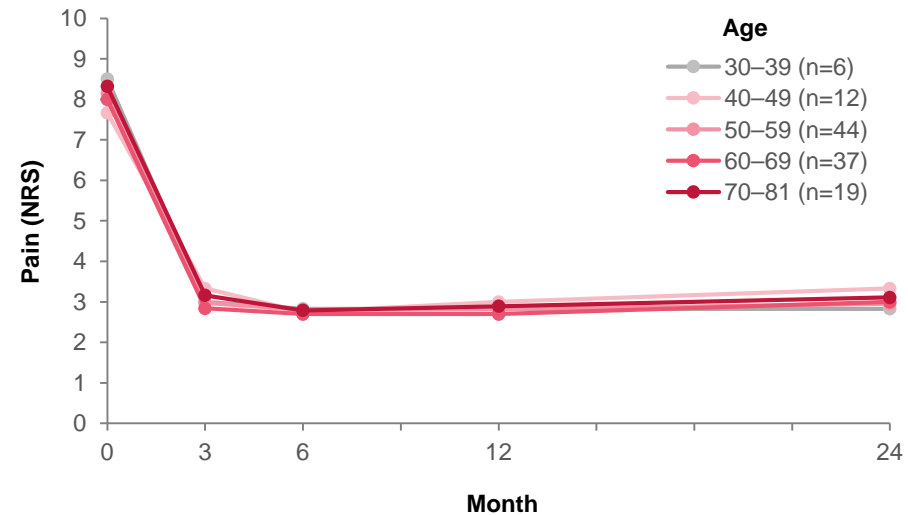**C**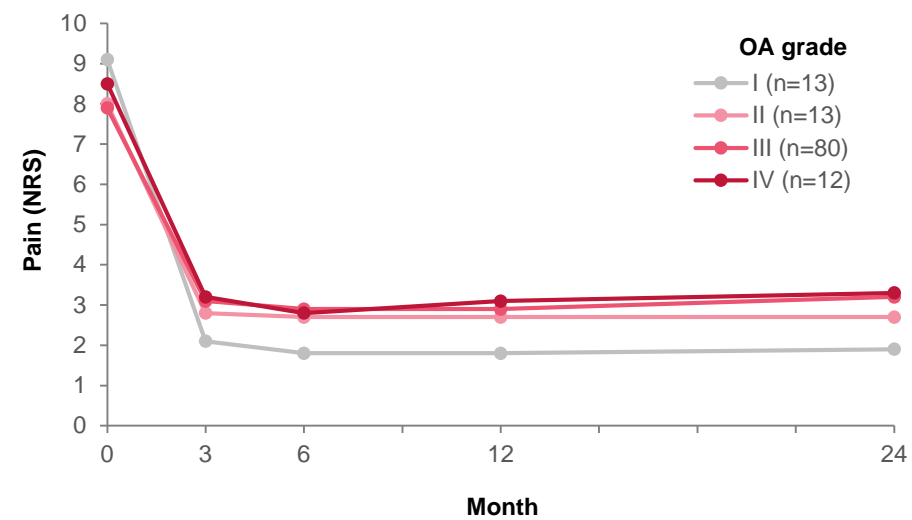**D**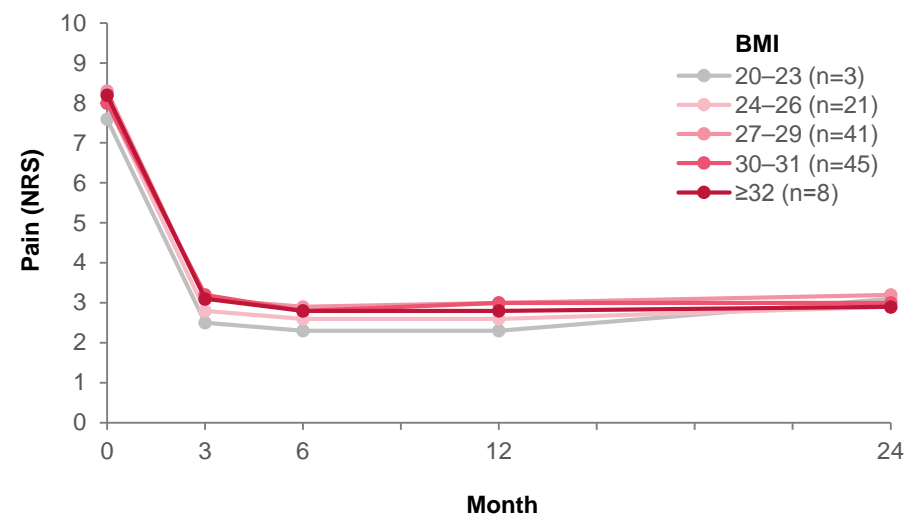

Supplement: S1 Fig — (PDF) [file pone.0145551.s001.pdf]
