# Supplementary material for: Treatment of Osteoarthritis of the Knee with a Combination of Autologous Conditioned Serum and Physiotherapy: A Two-Year Observational Study
Source: PLoS One. 2015 Dec 28;10(12):e0145551. doi: 10.1371/journal.pone.0145551 (PMC4692499; doi:10.1371/journal.pone.0145551)
Supplement: S1 Table — (DOCX) [file pone.0145551.s002.docx]

**S1 Table.** **Stratification of WOMAC global by pain (NRS) outcome**

| **Pain (NRS)** | **n** | **Baseline**  **Mean WOMAC global (range)** | **24 months**  **Mean WOMAC global (range)** | **% Mean reduction in**  **WOMAC global** |
| --- | --- | --- | --- | --- |
| 10 | 10 | 81.10 (76–90) | 35.80 (30–40) | 55.9 |
| 9 | 31 | 83.10 (75–90) | 34.39 (30–42) | 58.6 |
| 8 | 42 | 81.29 (76–89) | 35.21 (30–42) | 56.7 |
| 7 | 31 | 81.16 (75–90) | 35.87 (31–42) | 55.8 |
| 6 | 4 | 77.75 (75–81) | 34.25 (30–39) | 55.9 |
